# Continuous glucose monitoring, diabetes distress, and well-being in adults with type 1 diabetes: findings from a National Survey in Lithuania

**DOI:** 10.3389/fcdhc.2026.1708124

**Published:** 2026-03-13

**Authors:** Jurga Šuminienė, Rimantas Stukas, Virginija Gaigalaite, Dovilė Kriaučiūnienė, Natalja Istomina

**Affiliations:** 1 Department of Public Health, Institute of Health Sciences, Faculty of Medicine, Vilnius University, Vilnius, Lithuania; 2 Faculty of Medicine, Vilnius University, Vilnius, Lithuania; 3 Centre of Endocrinology, Vilnius University Hospital Santaros Klinikos, Vilnius, Lithuania; 4 Institute of Health Sciences, Faculty of Medicine, Vilnius University, Vilnius, Lithuania

**Keywords:** continuous glucose monitoring, diabetes distress, hypoglycemia confidence, type 1 diabetes, well-being

## Abstract

**Background:**

Continuous glucose monitoring (CGM) has transformed glycemic management in type 1 diabetes (T1D), yet its associations with general well-being remain heterogeneous.

**Objective:**

To examine associations between glucose monitoring modality and general well-being among adults with T1D in Lithuania and to explore relationships between diabetes distress, hypoglycemia confidence, and well-being, including within predefined higher-risk subgroups.

**Methods:**

A cross-sectional national online survey was conducted between December 2023 and May 2024 among 368 adults with T1D (171 using flash glucose monitoring [FGM] and 197 using continuous glucose monitoring [CGM]). Participants completed the WHO-5 Well-Being Index, Diabetes Distress Scale (DDS-17), and Hypoglycemia Confidence Scale. Multivariable logistic regression models were used to identify predictors of good well-being (WHO-5 ≥50) and high hypoglycemia confidence (≥3), adjusting for sociodemographic and clinical factors. Median duration of CGM use was 24 months (IQR 12–36).

**Results:**

Compared with FGM users, CGM users reported lower diabetes distress, higher hypoglycemia confidence, and higher median WHO-5 scores. After adjustment, CGM use was independently associated with high hypoglycemia confidence but not with good general well-being. Better glycemic stability (time in range >70%) and absence of recent acute events were independently associated with higher odds of good well-being. In selected higher-risk subgroups (unemployment, frequent non-severe hypoglycemia, and time in range <70%), CGM users more frequently reported good well-being; these findings represent cross-sectional associations.

**Conclusion:**

CGM use was associated with improved diabetes-specific emotional outcomes, particularly hypoglycemia confidence. Associations with general well-being appear to operate indirectly through glycemic stability and hypoglycemia-related factors. These findings support integrating CGM into comprehensive, patient-centered diabetes care models, particularly for individuals with elevated psychosocial vulnerability.

## 1 Introduction

Type 1 diabetes (T1D) requires lifelong insulin therapy and regular glucose monitoring to maintain glycemic control. In Lithuania, more than 7,500 adults live with T1D (1). Beyond metabolic management, T1D imposes a substantial psychological burden, including diabetes-related distress and fear of hypoglycemia, which negatively affect quality of life and daily functioning (2, 3). Contemporary diabetes care therefore increasingly integrates patient-reported outcomes alongside biomedical indicators.

Continuous glucose monitoring (CGM) has transformed diabetes management and is consistently associated with improved glycemic control and reduced severe hypoglycemia (4, 5). Evidence suggests that CGM may reduce diabetes-related distress and improve hypoglycemia-related confidence (6–8), although findings regarding generic well-being remain heterogeneous.

Notably, flash glucose monitoring (FGM) and real-time CGM differ functionally, as CGM provides continuous real-time data with automated alerts, whereas FGM requires active scanning. These differences may have implications for perceived safety and emotional outcomes.

Recent studies indicate that psychosocial outcomes associated with glucose monitoring technologies are context-dependent. Early initiation and longer duration of CGM use have been associated with reduced distress without adverse psychological consequences (9). However, benefits may vary across high-risk or socially vulnerable groups, where technology-related burden or burnout may coexist with clinical improvements (10, 11). Emerging evidence further suggests that emotional distress and hypoglycemia-related worry may influence engagement with diabetes technologies (12), and that diabetes-related distress remains a central determinant of quality of life, sometimes independent of technology use (13).

In Lithuania, state-funded CGM reimbursement for adults with T1D began in July 2022, creating an opportunity to evaluate real-world psychosocial associations at the national level. National data examining general well-being, diabetes distress, and hypoglycemia confidence in relation to glucose monitoring modality remain limited.

Therefore, this study aimed to examine associations between glucose monitoring modality (CGM versus flash glucose monitoring [FGM]) and general well-being (WHO-5) among Lithuanian adults with T1D. Additionally, we explored relationships between diabetes-specific distress, hypoglycemia confidence, and overall well-being, including within predefined higher-risk subgroups.

## 2 Materials and methods

### 2.1 Study design and participants

A cross-sectional national online survey was conducted between December 2023 and May 2024 in Lithuania. The survey link was disseminated via national patient organizations, diabetes-related forums, and social media groups for adults with T1D.

Eligible participants were adults (≥18 years) with self-reported T1D who completed the questionnaire independently. Of 512 initiated surveys, 368 were completed (completion rate 71.8%) and included in the final analysis.

### 2.2 Data collection

The questionnaire collected sociodemographic data (age, sex, education, employment status, cohabitation status), clinical characteristics (body mass index [BMI], diabetes duration), glucose monitoring modality (CGM or FGM), duration of CGM use (months; CGM users only), glycated hemoglobin (HbA1c, %), time in range (3.9–10.0 mmol/L), and acute and chronic diabetes-related complications.

Time in range (TIR) data were available for 250 participants.

### 2.3 Outcome measures

General well-being was assessed using the WHO-5 Well-Being Index (raw score 0–25, transformed to 0–100). Good well-being (primary outcome) was defined as WHO-5 ≥50, while scores ≤28 indicated poor well-being (14, 15).

Diabetes-related distress was measured using the 17-item Diabetes Distress Scale (DDS-17; mean score range 1–6), with a score ≥3 indicating high distress (16).

Hypoglycemia confidence was assessed using the 8-item Hypoglycemia Confidence Scale (mean score range 1–4), with a mean score ≥3 indicating high confidence (17).

### 2.4 Statistical analysis

Continuous variables were presented as medians (interquartile range [IQR]) and were compared using Mann–Whitney U tests due to non-normal distribution. Categorical variables were compared using χ² or Fisher’s exact tests, as appropriate. Spearman correlation coefficients were calculated to assess associations between WHO-5 scores, diabetes distress, and hypoglycemia confidence.

Multivariable logistic regression models were constructed to identify predictors of good well-being (WHO-5 ≥50) and high hypoglycemia confidence (≥3). Covariates were selected *a priori* based on clinical relevance and existing literature and included age, sex, employment status, BMI (≥30 kg/m²), recent acute events (past six months), diabetes duration, education, and glycemic indicators.

Education and diabetes duration were evaluated in preliminary models but did not materially alter effect estimates and were therefore omitted from the final presented models for parsimony; models including these variables yielded comparable results.

In the model predicting high hypoglycemia confidence, frequency of non-severe hypoglycemia (>1/week) was included as an additional clinically relevant covariate.

Insulin pump use was not included in multivariable models due to strong collinearity with CGM use and its very low prevalence among FGM users, which could have resulted in model instability.

To minimize collinearity, HbA1c and time in range (TIR) were not included simultaneously; TIR (>70%) was retained as a marker of glycemic stability.

In the full-cohort model, glucose monitoring modality (CGM vs FGM) was entered as a categorical variable. Because device duration data were available only for CGM users, CGM duration was evaluated separately in a secondary model restricted to CGM users, where it was entered as a continuous variable (per year of use).

Variables potentially representing mediators (e.g., device satisfaction) were not included in primary models to avoid overadjustment and preserve interpretation of clinically relevant associations.

Model assumptions were assessed and no major violations were identified. Adjusted odds ratios (ORs) with 95% confidence intervals (CIs) are reported. All tests were two-sided, and p-values <0.05 were considered statistically significant. Analyses were performed using SPSS version 24.0 (IBM Corp., Armonk, NY, USA).

## 3 Results

### 3.1 Study population characteristics

A total of 368 adults with type 1 diabetes (age range 18–76 years) participated in the study, including 171 FGM users and 197 CGM users (**Table 1**).

**Table 1:** Baseline characteristics of participants by glucose monitoring modality.

Sociodemographic characteristics	Total (n=368)	FGM (n=171)	CGM (n=197)	P-value
Sociodemographic characteristics				
Female sex, n (%)	216 (58.7)	100 (58.5)	116 (58.9)	0.9
Age, years, median (IQR)	40 (30-51)	45 (34-59)	37 (27.5-46.5)	0.001
Higher education, n (%)	249 (67.7)	97 (56.7)	152 (77.2)	0.001
Unemployed, n (%)	46 (12.5)	22 (12.9)	24 (12.2)	0.8
Living alone, n (%)	55 (14.9)	29 (17)	26 (13.2)	0.3
Diabetes history and treatment				
Diabetes duration, years, median (IQR)	13 (7-23)	14 (7-23)	13 (7-23)	0.8
Duration of CGM use, months (median, IQR)**	–	–	24 (12-36)	–
Insulin pump use, n (%)	44 (12)	1 (0.6)	43 (21.8)	0.001
BMI, kg/m², median (IQR)	24.1 (21.9-29.8)	24.7 (21.8-28.0)	24.1 (22-26.8)	0.15
Glycemic indicators				
Recent HbA1c, %, median, (IQR)	7.2 (6.8-8)	7.8 (6.7-8.4)	7.2 (6.8-8)	0.054
Time in range (3.9–10.0 mmol/L), median (IQR)*	68 (60-70)	59 (50-71)	69 (60-72)	0.005
Clinical burden				
Any acute event in past 6 months, n (%)†	66 (17.9)	52 (30.4)	14 (7.1)	0.001
Any chronic diabetes complication, n (%)‡	194 (52.7)	96 (56.1)	98 (49.7)	0.22

*TIR data available for 250/368 participants.

**Duration of CGM use available for CGM users only; not applicable to FGM users (p-value not calculated).

†Severe hypoglycemia and/or diabetic ketoacidosis in the previous 6 months.

‡Neuropathy, retinopathy, nephropathy, or history of diabetic foot ulcer.

CGM users were significantly younger than FGM users (median 37 vs 45 years, p=0.001) and more frequently had higher education (77.2% vs 56.7%, p=0.001). No significant differences were observed between groups in sex distribution, employment status, living situation, diabetes duration, or BMI.

Median duration of CGM use was 24 months (IQR 12–36). Duration data were available only for CGM users.

CGM users demonstrated higher median time in range (69% vs 59%, p=0.005) and a lower proportion of acute events in the previous six months (7.1% vs 30.4%, p=0.001). The prevalence of chronic diabetes complications did not differ significantly between groups.

### 3.2 Psychosocial outcomes

CGM users reported lower diabetes distress compared with FGM users (median DDS 2.2 vs 2.82, p=0.001), and a smaller proportion met criteria for high distress (27.9% vs 45.0%, p=0.001) (**Table 2**).

**Table 2:** Comparison of diabetes distress, hypoglycemic confidence and WHO-5 well-being in the groups with CGM and FGM.

Measure	FGM (n=171)	CGM (n=197)	P-value
Diabetes distress scale			
Diabetes distress score (median, IQR)	2.82 (2.1-3.6)	2.2 (1.7-3.1)	0.001
High distress (≥3), n (%)	77 (45.0)	55 (27.9)	0.001
Hypoglycemia confidence			
Total score, median (IQR)	2.55 (2.11-3.11)	3 (2.77-3.55)	<0.001
High confidence (≥3), n (%)	56 (32.7)	127(64.5)	<0.001
WHO-5 well-being			
WHO-5 score (0–100), median (IQR)	56 (44-68)	60 (48-76)	0.005
Good well-being (≥50), n (%)	114 (66.7)	140 (71.1)	0.3
Poor well-being (≤28), n (%)	25 (14.6)	8 (4.1)	0.002

DDS-17 range 1–6 (mean); high distress ≥3; Hypoglycemia Confidence Scale range 1–4 (mean); high confidence ≥3; WHO-5 raw score 0–25, transformed to 0–100; p-values from Mann–Whitney U or χ²/Fisher’s exact test.

Hypoglycemia confidence was higher among CGM users (median 3.0 vs 2.55, p<0.001), with 64.5% reporting high confidence compared with 32.7% of FGM users (p<0.001).

Median WHO-5 scores were modestly higher in CGM users (60 vs 56, p=0.005). However, the proportion of participants with good well-being (WHO-5 ≥50) did not differ significantly between groups (71.1% vs 66.7%, p=0.30). Poor well-being (WHO-5 ≤28) was less frequent among CGM users (4.1% vs 14.6%, p=0.002).

### 3.3 Associations between diabetes-specific measures and general well-being

In the overall cohort, WHO-5 scores were moderately negatively correlated with diabetes distress (Spearman r = −0.49, p<0.001) and moderately positively correlated with hypoglycemia confidence (r = 0.40, p<0.001).

### 3.4 Multivariable regression analyses

In the full-cohort model (Panel A), glucose monitoring modality (CGM vs FGM) was entered as a categorical variable (**Table 3**). After adjustment for age, sex, employment status, BMI ≥30 kg/m², recent acute events, and TIR >70%, CGM use was not independently associated with good well-being.

**Table 3:** Multivariable logistic regression models.

Panel A. Predictors of good well-being (WHO-5 ≥50), full cohort (N = 368)			
Predictors	Adjusted OR	95% CI	P-value
Age (per 10 years)	0.8	0.6-1.09	0.15
Female sex	0.57	0.3-1.2	0.14
Employed	1.7	0.9-3.3	0.06
BMI ≥30 kg/m^2^	0.4	0.2-0.8	0.019
Acute event (past 6 months)	0.12	0.02-0.8	0.033
TIR >70%	3.9	1.1-14	0.041
CGM use (vs FGM)	1.8	0.6-5.7	0.3

Panel B. Predictors of good well-being among CGM users (n=197)			
Predictors	Adjusted OR	95% CI	P-value
Age (per 10 years)	0.8	0.60–1.10	0.2
Female sex	0.4	0.2-0.8	0.016
Employed	1.2	0.4-3.4	0.7
TIR >70%	2.8	1.2-6.2	0.013
CGM duration (per year)	1.3	0.95-1.8	0.1

Panel C. Predictors of high hypoglycemia confidence (≥3)			
Predictors	Adjusted OR	95% CI	P-value
Age (per 10 years)	0.85	0.7-1.1	0.4
Higher education	2.4	1.3-4.0	0.003
Employed	1.3	0.7-2.5	0.4
Non-severe hypoglycemia >1/week	0.24	0.1-0.4	0.001
CGM use (vs FGM)	4.8	2.8-8.3	0.001

CGM vs FGM entered as categorical variable in Panel A; CGM duration available only for CGM users and analyzed separately (Panel B); HbA1c and TIR not included simultaneously due to collinearity; All models adjusted *a priori* for clinically relevant covariates.

Lower odds of good well-being were observed among participants with obesity (BMI ≥30 kg/m²) and those reporting acute events in the past six months, whereas TIR >70% was independently associated with higher odds of good well-being.

In the CGM-restricted model (Panel B), CGM duration was entered as a continuous variable (per year of use). Longer CGM duration showed a positive but non-significant association with good well-being after adjustment.

In Panel C, CGM use was independently associated with higher hypoglycemia confidence after adjustment for age, education, employment, and hypoglycemia frequency.

### 3.5 Higher-risk subgroup analyses

In selected higher-risk subgroups, higher proportions of CGM users reported good well-being compared with FGM users (**Table 4**). Among unemployed participants, 73.1% of CGM users reported good well-being compared with 44.8% of FGM users (p=0.034). Among individuals with TIR <70%, good well-being was reported by 60.0% of CGM users versus 26.7% of FGM users (p=0.001). Among participants reporting frequent non-severe hypoglycemia (>1/week), good well-being was more common in CGM users (85.4% vs 71.9%, p=0.047).

**Table 4:** Good well-being (WHO-5 ≥50) in selected higher-risk subgroups.

Subgroup	Total: n/N (%)	FGM: n/N (%)	CGM: n/N (%)	P-value
Unemployed (n=55)	32/55 (58.2)	13/29 (44.8)	19/26 (73.1)	0.034
Non-severe hypoglycemia >1/week (n=146)	117/146 (80.1)	41/57 (71.9)	76/89 (85.4)	0.047
TIR<70% * (n=155)	78/155 (50.3)	12/45 (26.7)	66/110 (60.0)	0.001

P-values compare CGM vs FGM within each subgroup (χ² or Fisher’s exact test).

Subgroups may overlap and analyses should be interpreted cautiously due to the cross-sectional design.

*TIR available for 250 participants.

TIR values were self-reported and available for 250 participants; therefore, analyses including TIR were based on available-case data.

These findings represent cross-sectional associations. No formal interaction testing was performed; subgroup differences should therefore be interpreted descriptively.

## 4 Discussion

This national survey provides real-world evidence on associations between glucose monitoring modality and psychosocial outcomes among adults with T1D in Lithuania. CGM users reported lower diabetes distress, higher hypoglycemia confidence, and higher median WHO-5 scores compared with FGM users. However, CGM use was not independently associated with good well-being (WHO-5 ≥50) after adjustment for clinical and sociodemographic factors.

The multivariable findings suggest that associations between CGM use and general well-being are likely indirect rather than direct. In our models, better glycemic stability (time in range >70%), absence of recent acute events, and lower BMI were independently associated with good well-being. CGM may act through these intermediate clinical and emotional factors—particularly hypoglycemia-related confidence—which are linked to broader emotional functioning. However, these hypothesized pathways cannot be formally tested within the present cross-sectional design. This pathway-based interpretation aligns with emerging evidence indicating that psychosocial effects of glucose technologies are context-dependent and influenced by engagement, distress levels, and support needs.

Flash glucose monitoring (FGM) and real-time CGM differ functionally. CGM systems provide continuous real-time glucose data with automated alerts for hypo- and hyperglycemia, whereas FGM requires active scanning and does not routinely include automated alerts. These functional distinctions may enhance perceived safety and situational awareness and likely contribute to the strong independent association observed between CGM use and higher hypoglycemia confidence.

Notably, rapid technological advances are progressively narrowing functional distinctions between flash and real-time glucose monitoring systems, as newer-generation sensors increasingly incorporate real-time data transmission and automated alert functionalities. The present findings reflect device use during an early phase of national CGM reimbursement implementation in Lithuania, when distinctions between device categories were more clearly defined. As glucose monitoring technologies continue to evolve, future research may benefit from focusing on specific device functionalities rather than traditional modality labels.

Hypoglycemia confidence was moderately correlated with WHO-5 scores, further supporting its relevance for overall emotional well-being.

Although median WHO-5 scores were higher among CGM users, the dichotomized outcome (WHO-5 ≥50) did not differ significantly between groups. This discrepancy may reflect reduced sensitivity resulting from dichotomization of a continuous measure, as well as the relatively generic nature of WHO-5 compared with diabetes-specific instruments. Reporting both continuous and categorical outcomes therefore provides a more comprehensive assessment of psychosocial impact.

In predefined higher-risk subgroups—including unemployment, frequent non-severe hypoglycemia, and suboptimal time in range—CGM users more frequently reported good well-being than FGM users. These analyses were clinically motivated but should be interpreted cautiously due to potential overlap between categories and the cross-sectional design. No formal interaction testing was performed; therefore, subgroup differences should be interpreted descriptively and considered hypothesis-generating. The consistency of directionality across clinically defined vulnerability markers may support the plausibility of these observations; however, confirmation in longitudinal and adequately powered studies is warranted.

Several limitations warrant consideration. First, the cross-sectional design precludes causal inference, and the observed associations should not be interpreted as effects. Second, recruitment through patient organizations and social media may have introduced self-selection bias and may limit generalizability, particularly to individuals with lower digital engagement. Direct measures of socioeconomic status and digital literacy were not available; therefore, residual treatment-related confounding cannot be excluded. Third, state reimbursement for CGM began only in 2022, resulting in relatively limited exposure duration and reduced variability in use; consequently, the study may have been underpowered to detect modest duration-dependent psychosocial associations. In addition, insulin pump use differed substantially between groups and was not included in multivariable models due to collinearity with CGM use; therefore, some residual treatment-related confounding cannot be entirely excluded.

Despite these limitations, this study represents one of the first nationwide assessments of psychosocial outcomes associated with CGM implementation in Lithuania using validated instruments. Overall, the findings suggest that CGM may be considered as part of comprehensive, patient-centered diabetes care models that incorporate structured education and psychosocial support, particularly for individuals at elevated psychosocial risk.

## 5 Conclusion

In this national survey of adults with type 1 diabetes in Lithuania, CGM use was associated with lower diabetes distress and higher hypoglycemia confidence compared with flash glucose monitoring. CGM was not independently associated with good general well-being after adjustment, suggesting that observed associations may operate indirectly through glycemic stability and hypoglycemia-related emotional factors. Associations appeared more pronounced in selected higher-risk subgroups; however, given the cross-sectional design and exploratory subgroup analyses, these findings should be interpreted cautiously. Longitudinal studies are needed to clarify temporal relationships and potential causal pathways.

Overall, the results support consideration of CGM as part of comprehensive, patient-centered diabetes care frameworks, particularly for individuals at elevated psychosocial vulnerability.

## Data Availability

The original contributions presented in the study are included in the article/**Supplementary Material**. Further inquiries can be directed to the corresponding author.
