# Supplementary material for: Continuous glucose monitoring, diabetes distress, and well-being in adults with type 1 diabetes: findings from a National Survey in Lithuania
Source: Front Clin Diabetes Healthc. 2026 Mar 13;7:1708124. doi: 10.3389/fcdhc.2026.1708124 (PMC13021413; doi:10.3389/fcdhc.2026.1708124)
Supplement: Supplementary file 1 [file DataSheet1.pdf]

Supplementary Table 1. Predictors for hypoglycemia confidence in T1D.

	Univariate analysis		Multivariate analysis*	
	Odds ratio, 95% CI	p	Odds ratio, 95% CI	p
Gender, women vs. men	0.75 (0.5–1.1)	0.18		
Age <20 y, increasing per 10 years	0.8 (0.68–0.96)	0.015		
Body mass index (kg/m2) >30	0.54 (0.24–1.2)	0.13		
Education: Higher Education	3.7 (2.2–6)	0.001		
Living alone	0.8 (0.4–1.4)	0.4		
Employed	2.1 (1.3–3.4)	0.004		
Place of residence: country vs city	0.8 (0.4–1.5)	0.5		
Duration of diabetes (<2, 2–4.9, 5–9.9, >10 years)	0.5 (0.3–0.8)	0.015		
CGM usage duration, per year	1.06 (0.96–1.3)	0.6		

Onset of diabetes ≤20 y, per 10 years	0.6 (0.5–0.8)	0.001	0.4 (0.2–0.9)	0.031
Diabetes-related complications, >1	0.5 (0.4–0.6)	0.001		
Diabetes-related critical times past 6 mo	0.13 (0.06–0.28)	0.001		
• Ketoacidosis	1.9 (0.6–6.6)	0.28		
• Severe hypoglycemia	0.06 (0.021–0.17)	0.001		
Non-severe hypoglycemia >1/wk	0.3 (0.2–0.5)	0.001	0.11 (0.04–0.4)	0.001
Non-severe hyperglycemia >1/wk	0.5 (0.3–0.7)	0.01		
HbA1c >7 mmol/l (7–7.9, 8–8.9, >9)	0.6 (0.5–0.8)	0.001	0.2 (0.07–0.4)	0.01
TIR (Time In Range) >70%	2.2 (1.2–4)	0.014		
Continuous glucose monitoring	3.7 (2.4–5.8)	0.001	-	0.002
Satisfied with the devices they used	5.4 (3–9.8)	0.001		
